# Pharmaceutical Strategy for Europe: Reflections on Public Health-Driven Drug Development, Regulation, and Policies

**DOI:** 10.3389/fphar.2021.685604

**Published:** 2021-04-28

**Authors:** Silvio Garattini, Yannis Natsis, Rita Banzi

**Affiliations:** ^1^ Istituto di Ricerche Farmacologiche Mario Negri IRCCS, Milan, Italy; ^2^ European Public Health Alliance, Brussels, Belgium; ^3^ Center for Health Regulatory Policies, Istituto di Ricerche Farmacologiche Mario Negri IRCCS, Milan, Italy

**Keywords:** pharmaceutical policies, incentives, drug development—clinical trials, European commission, added value, health policies, pharmaceutical medicine

## Introduction

Never before has the central role of medicines and other health care interventions for society been so recognized. Europe’s pharmaceutical sector is a major contributor to the European Union’s economy. According to the European Federation of Pharmaceutical Industries and Associations (EFPIA), it contributes with more than €110 billion to the EU trade balance and employs almost 800,000 people across Europe. In 2019, it invested an estimated € 37,500 million in R&D in Europe (European Federation of Pharmaceutical Industries and Associations, 2020). However, despite these investments, the drug market is not without some low-value drugs and alleged innovations, resulting in an excess of public and private spending that could be reduced in favour of other health-related activities. A survey by the independent scientific journal Prescrire found that only 10% of the new authorisations in 2019 presented a notable therapeutic advance (Prescrire, 2020), a view shared by the German Institute for Quality and Efficiency in Health Care (IQWiG) (Wieseler et al., 2019).

On 25 November 2020, in the midst of the COVID-19 pandemic, the European Commission adopted a new “Pharmaceutical strategy for Europe,” after intense rounds of consultation with stakeholders and the public between July and September 2020. This pharmaceutical strategy “aims to ensure the quality and safety of medicines, while boosting the sector’s global competitiveness” (European Commission, 2020a). It is a roadmap based on four specific pillars (Table 1), an inventory of actions. Some will make the legislative cut by the end of the current European Commission’s term. Unquestionably, this document offers an opportunity for thorough reflection on drug development, regulation, and policies.

**TABLE 1:** Pillars of the pharmaceutical strategy and key proposals.

Pillars of the pharmaceutical strategy for Europe (European Commission 2020a)	Proposals for health-driven drug development, regulation, and policies
Ensuring access to affordable medicines for patients, and addressing unmet medical needs	Shared agendas and reform of the incentive schemes, including intellectual properties, to address patients’ and public health needs, with an eye to equity and affordability
Supporting competitiveness, innovation and sustainability of the EU’s pharmaceutical industry and the development of high quality, safe, effective and greener medicines	Introduction of alternative paradigms to support competition and innovation to develop a more balanced system
Enhancing crisis preparedness and response mechanisms, diversified and secure supply chains, address medicines shortages	Promote coordination rather than competition, from R&D to manufacturing and distribution
Ensuring a strong EU voice in the world, by promoting a high level of quality, efficacy and safety standards	Assessment of added therapeutic value for marketing approval and transparent and accountable EU agencies

## Access to Affordable Medicines Addressing Unmet Medical Needs

The need for affordable medicine is a central issue of the Strategy, reflecting one of the top priorities in the European Health Commissioner’s mission statement (Von Der Leyen, 2019). The Strategy acknowledges that research investment does not necessarily focus on the greatest unmet needs, mainly because of limited commercial interest. The call for “research priorities aligned to the needs of patients and health systems” can be made concretely by promoting shared agendas that identify, prioritize, and achieve consensus on the research areas or questions of importance to stakeholders. Methods have been developed but should now become a standard approach of European and national research bodies. New types of incentives may be explored, for instance, in the field of rare diseases, given the substantial limitations of the current approach. The methodological quality of orphan medicinal product applications is generally poor, as well as the information about their harm-benefit profiles at the time of approval (Joppi et al., 2013). A recent analysis of 20 years of orphan drugs reported that average annual sales rose from €133m in 2001 to €723m in 2019 (Marselis and Hordijk, 2020). The European Commission itself looked at the orphan medicines introduced between 2001 and 2016 and estimated that the number of new orphan medicines that can be attributed to the EU Orphan Regulation was extremely low (12–16%), and the majority would probably have reached the market anyway (European Commission, 2020b). These orphans, however, had benefited from superfluous monopoly rights. The revision of the EU orphan legislation—rightly prioritized by the European Commission—should also increase the availability and affordability of these medicines in all EU Member States, promoting fair access that has been far from guaranteed to date.

New approaches should prioritize a critical review and a possible scale-back of intellectual property (IP)-based monopolies and exclusivities, and lay emphasis on not-for-profit business models. The EMA lists about 2,000 candidate orphan drugs that could be adopted by networks of academic and health research institutes and foundations, duly funded to develop them, and possibly achieve their licensing. This could also set in motion a new role for academic independent clinical research, more closely embedded in drug development and licensing, to directly provide answers to public health needs (Garattini and Chalmers, 2009).

A new paradigm will require strong governance and long-term commitments in funding and investments. It could also help re-establish the correct symmetry in public-private partnerships.

## Competitiveness, Innovation and Sustainability

The current pharmaceutical innovation and IP system has repeatedly shown its fault line, besides a failure in global health goals (Boulet et al., 2019). The COVID-19 crisis magnifies the need for a shift in this paradigm to allow rapid and widespread access to effective low-priced treatments. Any revision of the patent-based monopolies-exclusivities will not only have an impact on competition and public health expenditures in the EU, but could drive a global change that is pressing for lowest-income countries.

The Commission recently adopted an Action Plan on IP, reaffirming its importance for innovation and the competitiveness of the European industry (European Commission, 2020c). Several actions relevant for access to medicines and global public health are included, illustrating the willingness to develop a more balanced system (Ellen’t Hoen, 2020). Voluntary pooling and licensing of patents may be applicable not just in emergencies; it should become an option whenever a public health issue arises. This would also help increase the production of old but effective products and reduce drug shortages. It would be even more obvious to abandon the supplementary protection certificates that are applied differently in the Member States and pose impossible obstacles to the development and licensing of generics and biosimilars. The Action Plan on IP calls on Member States to put in place procedures for issuing compulsory licenses when necessary, i.e, allowing the production of patented products without the consent of the patent owner (World Trade Organisation, 2020). Though some may view compulsory licensing in the EU as a radical measure, it has been recently endorsed by such unexpected observers as the Financial Times (Financial Times, 2021).

Experience with the EU joint procurement for COVID-19 vaccines and therapeutics (as for remdesivir) showed that EU countries can exchange information, negotiate drug prices jointly and even buy pharmaceutical products together. The publication of redacted versions of contracts signed between pharma and EU is an important precedent for good governance and transparency. Joint procurement procedures aim to ensure that participating countries have uninterrupted supply and increase the bargaining power of the public counterpart in price negotiations. However, it is hard to assess its actual impact because of the confidentiality of price negotiation. To be accountable, any co-operation between public authorities and industry should be based on full transparency on agreements and price definitions, including clinical trial costs and other determinants of pricing along the value chain from laboratory to patient (World Health Organization, 2019).

## Crisis Preparedness and Response Systems

The pandemic has highlighted the need to strengthen the EU’s capacity to respond to health issues. A major step will be the establishment of an EU Health Emergency Response Authority to anticipate and monitor threats but also to identify “promising and innovative countermeasures” (European Commission, 2020a). The Commission is currently exploring its functions and possible structure, working on a proposal that will be openly discussed and presented to the Member States and European Parliament in the second half of 2021.

Though focused on emergencies, this authority may promote overall harmonization and effective public-driven coordination of the EU pharmaceutical environment, from R&D investments to manufacturing and distribution.

As for research, networks already in place have the potential to assist researchers in setting up multinational studies and are the ideal collector of the best expertize in the Union. The COVID-19 crisis dramatically showed how innovative trial designs to evaluate multiple interventions simultaneously can produce rapid and reliable answers (Gaba and Bhatt, 2020). Research institutions and public hospitals should be firmly encouraged and duly funded to work collaboratively rather than competitively, avoiding fragmenting and the conduction of small national monocentric trials, that are often inconclusive and lead to waste of research. Large consortia can promote shared approaches to data measurement and collection, forming the bases for better exploitation of new technologies for drug development and assessment. Health digitalization and open data are the pillars of this change.

Besides research, the pharmaceutical environment needs to ensure the proper preparation and distribution of medicinal products, as for instance to address medical shortages. The current system dominated by the private sector often shows drawbacks. Measures to manage and prevent shortages of medicines include monitoring, facilitated regulatory procedures, and legal provisions to impose export bans so as to maintain supply reserves. Stronger actions may include a mechanism for building up or converting facilities in emergency situations.

## EU Leadership in Quality, Efficacy and Safety Standards

The Strategy mentions that the revision of the pharmaceutical legislation should include new methods of evidence generation and assessment, as well as analysis of big and real-world data to support the development, authorization and use of medicines. The Strategy clearly re-affirms the pivotal role of robust clinical trials with suitable comparators for the authorization of innovative medicines, without any intention of lowering the evidence standards (European Commission, 2020a). However, there is an opportunity here to revise the current criteria for the approval of new medicines. Although “quality, efficacy and safety” are fundamental characteristics for any drug on the market, health systems, clinicians and patients need to know if a new drug is better or worse than the drugs already available for the same indications (Garattini, 2021). Including the concept of “added therapeutic value” in the legislation would boost knowledge on the actual value of new drugs, thus setting the bases for fairer prices and appropriate use in clinical practice. Furthermore, the added therapeutic value would require the assessment of a real advantage, such as a reduction in symptoms or mortality or improvement of quality of life, thus boosting more relevant clinical studies in the interests of patients and public health. A more pronounced shift in the paradigm of drug development could be achieved if regulatory authorities were to require at least one pivotal phase III trial to be conducted by independent scientific organisations to support the marketing authorization.

Strengthening the EU voice in the global pharma endeavour should involve the recognition of the accountability and independence of EU agencies. The EMA has undoubtedly raised its level of transparency and openness to dialogue with society. An important area of better transparency is that of the scientific advice. Implementing the European Ombudsman’s recommendations on how to strengthen transparency in the pre-submission activities in the assessment of new drugs goes in the right direction (European Ombudsman, 2019). However, it is the essence of the EMA funding mechanism to cast a shadow. Almost the whole EMA budget comes from fees and charges from industry, 86% in 2020 (European Medicines Agency, 2020). Alternative funding schemes should be explored, as for instance direct support from the Commission funded by tax-driven systems. The public’s perception of independence and integrity is of the utmost importance and the EMA should actively dispel any fears about regulatory capture.

## Discussion

The Strategy is a pivotal document. It sets the basis for important improvements in the pharma field and responds to needs and requests widely shared by many health professionals and services. It is also broad in scope, with important references to important challenges such as the EU’s economy sustainability, Europe’s Beating Cancer Plan, and the European Digital Strategy.

We have discussed four main areas where efforts should be concentrated. First, legislative action to reform the incentive schemes to address patients’ and public health needs, with an eye to equity and affordability. Second, the introduction of alternative paradigms supporting competition and innovation. Third, efforts to promote coordination rather than competition in the EU. Last, the maintenance of high standards for drug approval by transparent and accountable decision-making bodies.

Moving from auspices to facts will not be cost-free. Any change to the rules of the game in a field like pharma will affect many stakeholders and interests. The medical and research communities should reinforce and suggest to politicians measures that could shape health-driven drug development, regulation, and policies.

## References

[B1] Boulet P. Garrison C. Ellen’t Hoen E. (2019). European Union Review of Pharmaceutical Incentives: Suggestions for Change. Available at: https://medicineslawandpolicy.org/wp-content/uploads/2019/06/MLP-European-Union-Review-of-Pharma-Incentives-Suggestions-for-Change.pdf (Accessed March 18, 2021)

[B2] Ellen’t Hoen E. (2020). Some Surprises in the European Commission’s New Intellectual Property Strategy. Available at: https://medicineslawandpolicy.org/2020/12/some-surprises-in-the-european-commissions-new-intellectual-property-strategy/ (Accessed March 18, 2021)

[B3] European Commission (2020a). Communication from the Commission to the European Parliament, the Council, the European Economic and Social Committee and the Committee of the Regions. A Pharmaceutical Strategy for Europe. Available at: https://ec.europa.eu/health/human-use/strategy_en (Accessed March 18, 2021)

[B4] European Commission (2020b). Evaluation of the Orphan and Paediatric Regulation. Available at: https://ec.europa.eu/health/sites/health/files/files/committee/ev_20200312_791_en.pdf (Accessed March 18, 2021)

[B5] European Commission (2020c). Commission Adopts Action Plan on Intellectual Property to Strengthen EU's Economic Resilience and Recovery. Available at: https://ec.europa.eu/commission/presscorner/detail/en/IP_20_2187 (Accessed March 18, 2021)

[B6] European Federation of Pharmaceutical Industries and Associations (2020). The Pharmaceutical Industry: a Key Asset to Medical Progress and the European Economy. Available at: https://efpia.eu/publications/downloads/efpia/2020-the-pharmaceutical-industry-in-figures/ (Accessed March 18, 2021)

[B7] European Medicines Agency (2020). Available at: https://www.ema.europa.eu/en/about-us/how-we-work/governance-documents/funding (Accessed March 18, 2021)

[B8] European Ombudsman (2019). Ombudsman Inquiry Strengthens Transparency and Objectivity of EMA's Assessment of New Medicines. Available at: https://www.ombudsman.europa.eu/en/case/en/49999 (Accessed March 18, 2021)

[B9] Financial Times (2021). Coronavirus Must Not Destroy an Open World Economy. Available at: https://www.ft.com/content/4a3bf282-701c-11ea-9bca-bf503995cd6f (Accessed March 18, 2021)

[B10] Gaba P. Bhatt D. L. (2020). The COVID-19 Pandemic: a Catalyst to Improve Clinical Trials. Nat. Rev. Cardiol. 17, 673–675. 10.1038/s41569-020-00439-7 32873977 PMC7461742

[B11] Garattini S. Chalmers I. (2009). Patients and the Public Deserve Big Changes in Evaluation of Drugs. BMJ 338, b1025. 10.1136/bmj.b1025 19336489

[B12] Garattini S. (2021). Quality, Efficacy, Safety—It is not Enough!. Eur. J. Clin. Pharmacol. 10.1007/s00228-021-03110-3 33687517

[B13] Joppi R. Bertele’ V. Garattini S. (2013). Orphan Drugs, Orphan Diseases. The First Decade of Orphan Drug Legislation in the EU. Eur. J. Clin. Pharmacol. 69, 1009–1024. 10.1007/s00228-012-1423-2 23090701

[B14] Marselis D. Hordijk L. (2020). From Blockbuster to “Nichebuster”: How a Flawed Legislation Helped Create a New Profit Model for the Drug Industry. BMJ 370, m2983. 10.1136/bmj.m2983 32727745

[B15] Prescrire (2020). Drugs in 2019: a Brief Review. Prescrire Int. 40, 146

[B16] Von Der Leyen U. (2019). Mission Letter. December 2019. Available at: https://ec.europa.eu/commission/commissioners/sites/comm-cwt2019/files/commissioner_mission_letters/mission-letter-stella-kyriakides_en.pdf (Accessed March 18, 2021).

[B17] Wieseler B. McGauran N. Kaiser T. (2019). New Drugs: where Did We Go Wrong and what Can We do Better?. BMJ 366, l4340. 10.1136/bmj.l483710.1136/bmj.l4340 31292109

[B18] World Health Organization (2019). World Health Assembly, 72. Improving the Transparency of Markets for Medicines, Vaccines, and Other Health Products. Available at: https://apps.who.int/iris/handle/10665/329301 (Accessed March 18, 2021)

[B19] World Trade Organisation (2020). Overview: the TRIPS Agreement. Available at: https://www.wto.org/english/tratop_e/trips_e/intel2_e.htm (Accessed March 18, 2021)

